# Genetic and Genomic Literacy of Healthcare Providers Treating Anorexia Nervosa in the United States: A Mixed Methods, Cross‐Sectional Study

**DOI:** 10.1002/brb3.70441

**Published:** 2025-03-31

**Authors:** Sarah Ramsay, Kendra Allison, Heide S. Temples, William C. Bridges, Sara Sarasua

**Affiliations:** ^1^ Healthcare Genetics Program, School of Nursing Clemson University Clemson South Carolina USA; ^2^ School of Nursing College of Behavioral, Social and Health Sciences, Clemson University Clemson South Carolina USA; ^3^ School of Mathematical and Statistical Sciences Clemson University Clemson South Carolina USA

**Keywords:** anorexia nervosa, genetic and genomic literacy, genetic and genomic testing, survey

## Abstract

**Background:**

Genetic testing has the potential to transform the prevention, treatment, and management of anorexia nervosa (AN) as it has for other conditions. However, healthcare providers require the knowledge and openness to implement genetic testing effectively.

**Objectives:**

This study had two main objectives, first, to determine the genomic literacy of those treating AN in the United States and second to assess the viewpoints of these healthcare providers on genetic testing and research, and the influence of genetics on AN.

**Methods:**

A mixed methods approach combining the GKnowM, a validated genomic literacy tool, Likert‐like statements and thematic analysis of free‐text responses was used. Participant consent, dissemination of the survey, and response collection were performed through Qualtrics.

**Results:**

Participant's average GKnowM score was 19.6 (SD = 2.8) on a scale of 0–26 (75% correct). Positive correlations were identified between GKnowM score and responses to questions about the influence of genetics on AN and the importance of genetics research, and negative correlations were found between age and years in practice and views on the current value of genetic testing. In addition, participants communicated a need for more genetics learning opportunities, and the challenge of accessing and paying for quality AN treatment in the United States.

**Discussion:**

The results of this study indicate a need for targeted genetics and genomics learning opportunities for healthcare providers. Improving genomic literacy has the potential to positively influence attitudes toward genetic research and testing and empower healthcare providers to engage in productive and scientifically sound discussions with their patients and society as a whole.

## Graphical abstract

Genetic testing has the potential to transform the prevention, treatment, and management of anorexia nervosa as it has for other conditions. However, healthcare providers require the knowledge and openness to implement genetic testing effectively.

We assessed the genomic literacy of those treating anorexia nervosa in the United States and their viewpoints on genetic testing and research, and the influence of genetics on anorexia nervosa.

Our findings indicate a need for targeted genetics and genomics learning opportunities for healthcare providers.

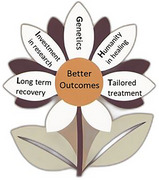

## 1 Introduction

Anorexia nervosa (AN) is a severe and debilitating disorder characterized by a persistent refusal of adequate food intake to maintain a normal body weight, often accompanied by an intense fear of gaining weight, a disturbance in the ability to accurately perceive one's own size and shape, and notable impairment to quality of life (American Psychiatric Association 2013). The standardized mortality ratio (SMR) estimates the likelihood of those in a given study population dying as compared to a reference population (Kelsey 2008). SMR estimates indicate that AN has between 6 and 16 times excess mortality, making it one of the deadliest psychiatric illnesses, second only to opioid use disorder (van Eeden et al. 2021; van Hoeken and Hoek 2020). Of those who do survive, it is estimated that approximately 20% go on to develop a more chronic illness, often referred to as severe and enduring (longstanding) anorexia nervosa (SE‐AN) (Eddy et al. 2017; Steinhausen 2002). While currently available treatments do help many patients with eating disorders, there are no FDA‐approved pharmaceuticals for the treatment of AN and progress has been slow in improving the effectiveness of current therapies overall and for SE‐AN in particular. The average inpatient hospitalization cost for eating disorder treatment was over $19,000 per admission in 2016 (Owens et al. 2019). The same year, the cost of residential care needed for weight restoration was estimated to be as much as $168,000, and those costs have no doubt increased since then (Guarda et al. 2017). Lack of effective treatments combined with high social and economic costs have resulted in a crisis in AN care in the United States (Kaye and Bulik 2021).

Contrary to popular belief, the overall incidence rate for AN has remained relatively stable as a whole in recent decades (4% female lifetime–0.3% male lifetime) (van Eeden et al. 2021; Smink et al. 2012). Symptomology and presentation of AN have evolved along cultural lines. However, it is not simply a manifestation of modern cultural and social pressures; accounts of deliberate self‐starvation date back to the beginning of written history (Bemporad 1996).

The etiology of AN is complex and includes both biological and environmental factors. Research indicates that genetic factors play a significant role in the development of AN as well as its persistence. Data gathered through twin and family studies estimate that the heritability of AN is between 28% and 83%, with studies using narrower definitions and inclusion criteria consistently yielding higher estimates (Martínez et al. 2022; Steiger and Booij 2020; Bulik et al. 2019; Dellava et al. 2011; Klump et al. 2001; Kortegaard et al. 2001; Wade et al. 2000).

Currently, there are no objective biomarkers capable of diagnosing AN, identifying those at risk for developing AN, or predicting its progression. As a result, AN has a propensity for misdiagnosis due to its associated medical complications and has been referred to as the “great pretender” (Andersen 2004). One example is the autosomal recessive metabolic disorder citrin deficiency (CD), which is caused by pathogenic variants of the *SLC25A13* gene, mimics AN and has been misdiagnosed as such (Takeuchi et al. 2015). Similar case reports for other genetic conditions have also been published (Dalle Grave et al. 2022; Feeney and Buell 2018; de Alves Pereira Carvalho Saraiva et al. 2022). In the future, genetics hold promise in providing more objective measures, thereby decreasing misdiagnosis and improving treatment outcomes.

The past quarter century has seen rapid advancements in genome analysis including genome‐wide association studies (GWAS). In 2019, the largest AN GWAS to date was published, identifying eight AN‐associated risk loci and genetic associations between AN and several metabolic factors including BMI supporting the reconceptualization of AN as a metabo‐psychiatric disorder (Watson et al. 2019). In that study, the GWAS indicated that the polygenic risk score (PRS) accounted for only 1.7% of phenotypic variation meaning that there were numerous additional AN‐associated genetic loci yet to be discovered. Since that time, several studies have examined the clinical utility of PRS for AN (Curtis et al. 2023; Yilmaz et al. 2023; Johansson et al. 2022). PRS, also called polygenetic scores (PGS) estimate an individual's genetic risk or predisposition toward a trait (Palla and Dudbridge 2015). In 2023, Yilmaz et al. were able to predict obsessive compulsive disorder (OCD), anxiety, and eating disorder phenotypes, including compulsive exercise, using AN, OCD, and AN/OCD PGS (Yilmaz et al. 2023). This was followed in 2022, with Johansson et al. (2022) providing evidence for the potential clinical utility of PRS for AN, when they associated AN PGS and BMI PGS with eating disorder severity. In 2023, Curtis et al. (2023) provided further support for AN PRS clinical utility when they found that the AN PRS utilized was significantly associated with eating disorder outcome. The authors for all these studies point out that with larger GWAS sample sizes, additional AN‐associated risk variants will be identified, further strengthening the clinical utility of AN PRS in predicting susceptibility to development of AN as well as AN severity. In short, PRS and other genetic assessments have the potential to provide a level of biologically based diagnostic and therapeutic precision at the individual level currently not available to those with AN.

Direct‐to‐consumer genetic testing (DTC‐GT) has greatly expanded the general public's access to their own genetic data. However, the lack of inclusion of an accessible and knowledgeable healthcare provider (HCP) in the process can be problematic (Levitt 2001; Oh 2019). Furthermore, even when HCPs are involved, they often lack sufficient genomic^1^ literacy to effectively help their patients interpret results (Ong et al. 2022; Martins et al. 2022).

Genetics and genomics information is complex and challenging to interpret, even for those well trained in the area. Genetics and genomics technologies are advancing at a remarkable pace, with the resulting data providing new insights on a nearly daily basis. In 2018, the United States Food and Drug Association (US FDA) began approving DTC genetic testing (U.S. Food & Drug Administration 2018). This, coupled with a dearth of certified genetic counselors, is placing increased responsibility for interpreting and communicating ever increasing amounts of genetic and genomics information on nongenetic professionals (Hoskovec et al. 2018; Dougherty et al. 2016; Campion et al. 2019). Genomic literacy, from a healthcare perspective can be defined as “the capacity to obtain, process, understand, and use genomic information for health‐related decision‐making” (Hurle et al. 2013). Numerous studies have pointed out genomic literacy deficits throughout the healthcare community, from medical school students to practicing clinicians (Ha et al. 2018; Alotaibi and Cordero 2021; Swandayani et al. 2021; Chow‐White et al. 2017; Wright et al. 2019). Ascertaining the current genomic literacy of those treating AN is a critical first step in the development of effective educational tools designed specifically for the needs of this group of HCPs^2^. Genomic literacy is not only important for physicians; a solid understanding of clinical genetics and genomics is vital to all HCPs.

HCPs' views on the degree to which genetic variation contributes to AN are also important. Data collected for other neurodevelopmental and psychiatric disorders, including schizophrenia, autism spectrum disorder, depression, and bipolar disorder, indicate that though genetic testing and counseling have the potential to be helpful, they are still underutilized in practice (Hippman et al. 2016). The stigma associated with AN is heightened by the misperception of the general public and many HCPs that AN is a voluntary illness and largely based on societal influences (Crisafulli et al. 2008). Knowing where this bias is based, as well as other barriers, could help develop educational campaigns and best practices to ensure that genetic testing is implemented in AN care where warranted, both now and in the future.

The objectives of this study were twofold. First, to obtain an overview of the genomic literacy of HCPs working with AN in the United States. The second objective was to assess HCPs' attitudes toward the impact of genetics on AN and the current and future utility of genetic testing in AN treatment where warranted. Participant demographics were also collected to assess any correlation between genomic literacy, views, and years of experience in treating the population, location (state, urban, suburban, and rural), gender, educational background, and self‐assessed familiarity with genetic testing.

## 2 Methods

### 2.1 Research Design

This study used a mixed methods approach. A cross‐sectional online survey of HCPs treating AN in the United States, consisting of a genomics literacy assessment combined with a series of questions designed to assess perceptions and attitudes toward the contribution of genetics to AN and the potential utility of genomic testing was used. The study was developed and distributed through Qualtrics. Demographics data were requested, and to ensure that participants had the opportunity to provide further feedback, a free text box was also included. Any HCP treating AN in the United States was eligible. All respondents gave informed consent before participating in the study. Participants were informed that their personal information and responses would be kept private and confidential (see Supporting Information for survey and informed consent). The survey was active from June 10 to September 30, 2024. The protocol was reviewed by the Clemson University Institutional Review Board IRB2024‐0281 and determined to be exempt.

### 2.2 Instruments Used

The validated 26‐item GKnowM genomics knowledge scale was used to assess genomic literacy. Development and validation of the GKnowM by Linderman et al. (2021) is described in detail elsewhere. To assess attitudes toward genetics and genetic testing with regard to AN, six Likert‐like (Likert 1932) questions were developed. The study was piloted by five Clemson Healthcare Genetics graduate students before launch to assess content, clarity, and flow.

Thematic analysis of the open‐text responses was conducted according to the Braun and Clarke method (Braun and Clarke 2006). First, all open‐text responses were downloaded from Qualtrics, without editing or contextualizing. All responses were reviewed in their entirety to gain familiarity with the data as a whole. Second, initial concept codes (“category that is used to describe a general feature of data”; Gibson and Brown 2009) were generated by two reviewers (Sarah Ramsay and Kendra Allison) to group pieces of information. Third, the codes were further evaluated for similarities, differences, and relationships to generate themes. In Steps 4 and 5, these themes were further analyzed, reviewed, and defined. Finally, in Step 6, the defined themes are reported.

### 2.3 Recruitment

Publicly available contact information for HCPs was collected from two treatment finder websites: FindEDHelp, and the Anorexia Nervosa and Associated Disorders (ANAD) site. All HCPs with email addresses stating that they treat AN were included. In addition, links to the survey were posted on a private eating disorder treatment provider Facebook page, and business cards with the study title and QR code were distributed at an eating disorder conference. Snowball sampling was also used as participants had the opportunity to refer others to the study. Participants included the following: AMFT: Associate Marriage and Family Therapist; APCC: Associate Professional Clinical Counselor; DO: Doctor of Osteopathic Medicine; LAC: Licensed Associate Counselor; LCSW: Licensed Clinical Social Worker; LD: Licensed Dietician; LDN: Licensed Dietitian Nutritionist; LMFT: Licensed Marriage Family Therapist; LMHC: Licensed Mental Health Counselor; LPC: Licensed Professional Counselor; MD: Medical Doctor; PhD: Doctoral of Philosophy; PsyD: Doctoral of Psychology; RD: Registered Dietician; RDN: Registered Dietician Nutritionist; RN: Registered Nurse. “Other” group consisted of: LICSW: Licensed Independent Clinical Social Worker; Clinical psychology PhD student; MSW: Master of Social Work; APSW: Advanced Practice Social Worker.

### 2.4 Quality Control

CAPTCHA (Completely Automated Public Turing Test to tell Computers and Humans Apart) verification was used to help ensure that respondents were human. Qualtrics ExpertReview was used to check the overall quality of the responses. This review includes flagging of respondents who finished unusually fast or are suspected of being bots, straightlining (providing the same answers down a matrix table), completion rate, and unanswered questions. There were no flagged responses for those completing the survey.

### 2.5 Data and Statistics

#### 2.5.1 GKnowM Literacy Score

Each question answered correctly was given a score of one, thus the range of possible results was 0–26. Chance level: Chance level for the literacy portion of the study, defined as the number of questions that a person most likely could have answered correctly by chance (assuming a binomial distribution) was [(23) × (1/4) + (3) × (1/3)] = 6.5.

#### 2.5.2 Views Assessment

Each views question answer was given a numerical score ranging from 1 to 5 (see Figure 2).

#### 2.5.3 Statistical Analysis

Categorical nominal data: provider type, gender, state and location of practice, and insurance type

Categorical ordinal data: age group, length of time treating AN group, familiarity, and Likert views responses

Quantitative discrete data: GKnowM literacy scores

ANOVA was used to assess any relationships between categorical nominal data and mean literacy scores. Kendall's tau‐b (*τ*_b_) correlation coefficient was used to measure the strength and direction of the relationships between Categorical ordinal data: age group, years treating patients with AN group and Categorical ordinal data: Likert‐like statement views and familiarity responses. *τ*_b_ correlation coefficient was also used to measure the strength and direction of the relationships between categorical ordinal data: age group, years of treating patients with AN group, Likert‐like statement views, and familiarity responses and quantitative discrete data: literacy scores. Microsoft Excel was used to conduct ANOVA and generate means, and standard deviations for response groups. IBM SPSS Statistics software was used for *τ*_b_ correlation assessments.

## 3 Results

### 3.1 Participant Response Rates and Demographics

Emails were sent to 795 US email addresses acquired as explained in the methods section, and 127 responded, resulting in a 16% response rate for emails. An additional 18 responded by accessing an anonymous link or QR code. Table 1 provides an overview of participant demographics. The majority of respondents were female (93.0 %). The respondents were located in 35 states (Table S1) with the majority (62.8%) working in a suburban setting. Most (42.7%) respondents indicated that they did not accept any form of insurance. The most common age range of participants was 35–44 years (32.6%), with most (35.2%) being in practice for more than 15 years. Only 1.4% had less than 1 year of experience.

**TABLE 1:** Participant information.

Demographic	Group	Percent (*N*)
Age		Total responses: 144
	< 25	0 (0)
	25–34	25.0 (36)
	35–44	32.6 (47)
	45–54	21.5 (31)
	55–64	11.8 (17)
	65–74	7.6 (11)
	> 74	1.4 (2)
Gender		Total responses: 142
	Female	93.0 (132)
	Male	3.5 (5)
	Nonbinary/third gender	2.8 (4)
	Prefer not to say	0.7 (1)
Length of time treating anorexia nervosa		Total responses: 145
	< 1	1.4 (2)
	1–5 years	20.0 (29)
	6–10 years	30.3 (44)
	11–15 years	13.1 (19)
	> 15 years	35.2 (51)
Healthcare provider type		Total responses: 145
	LPC, LMHC, LCSW, LMFT, LAC, AMFT, APCC	42.1 (61)
	RD, RDN, LD, LDN	39.3 (57)
	PhD, PsyD	11.7 (17)
	MD, DO	4.1 (6)
	RN	0.7 (1)
	Other	2.1 (3)
Currently offering genetic testing		Total responses: 145
	Yes	2.1 (3)
	No	97.9 (142)
Payment accepted		Total responses: 143
	Both private and public insurance	18.2 (26)
	Private insurance only	39.2 (56)
	Does not accept private or public insurance	42.7 (61)
Practice community location		Total responses: 145
	Rural	2.8 (4)
	Online only	12.4 (18)
	Urban	22.1 (32)
	Suburban	62.8 (91)

*Note*: “Other” group consisted of: LICSW: Licensed Independent Clinical Social Worker, Clinical psychology PhD student, MSW: Master of Social Work, and APSW: Advanced Practice Social Worker.

Abbreviations: AMFT, Associate Marriage and Family Therapist; APCC, Associate Professional Clinical Counselor; DO, Doctor of Osteopathic Medicine; LAC, Licensed Associate Counselor; LCSW, Licensed Clinical Social Worker; LD, Licensed Dietician; LDN, Licensed Dietitian Nutritionist; LMFT, Licensed Marriage Family Therapist; LMHC, Licensed Mental Health Counselor; LPC, Licensed Professional Counselor; MD, Medical Doctor; *N*, number of responders; PhD, Doctoral of Philosophy; PsyD, Doctoral of Psychology; RD, Registered Dietician; RDN, Registered Dietician Nutritionist; RN, Registered Nurse.

Participants were also asked to assess their familiarity with genetic testing. The majority of participants (80.7%) indicated that they were “moderately” or “slightly” familiar with genetic or genomic testing. Another 13.1% indicated they were “not at all familiar,” and 6.2% were “very” or “extremely” familiar with genetic or genomic testing.

### 3.2 Genomic Literacy

**FIGURE 1:**
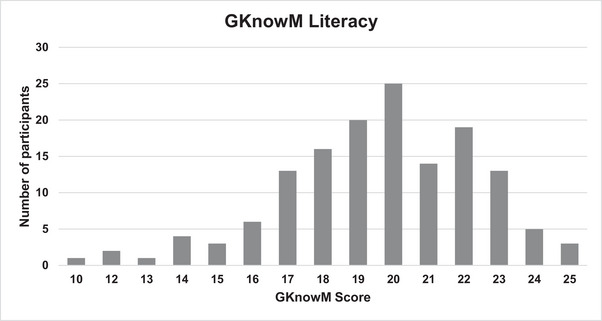
Overall genetic literacy scores. The mean score was 19.6 (SD = 2.8) (75% correct) on a scale of 0–26; *N* = 145.

The 26‐question GKnowM, developed by Linderman et al., was used to assess genomic literacy. This validated tool was developed to serve as an assessment of genomic literacy for a broad audience, including HCPs (Linderman et al. 2021). The chance level for the study, which is the number of questions that a person could have answered correctly by chance, was 7. A total of 145 participants completed the entire GKnowM assessment (26 questions). Figure 1 provides a graphical representation of scores for these participants. The mean score was 19.6 (SD = 2.8) on a scale of 0–26 (75% correct). No participants received a score below the chance level, and no participants answered all questions correctly. The lowest score was 10 of 26 and the highest, 25 of 26. The percent correct for each question was also calculated (Table S2). Question 8 had the highest percentage correct (100%) and Question 20 had the lowest percentage correct (34%).

Scores from this study were compared to those for the various groups studied by Linderman et al. (2021) by using the T‐score conversion presented in tab. 4 of that publication and the scores presented in tab. S5 of that publication. The calculated GKnowM T‐score mean for all participants in the current study was 58.3 (SD = 4.3) which was higher than the highest‐scoring HCP group assessed by Linderman et al. (*Health Care Provider Works with Genetic*/*Genomic Tests of Technologies* [57.7, SD = 13.8]), but lower than the *Undergraduate Students Enrolled in Human Genome Analysis Course* group after completing the course (62.6, SD = 2.9).

### 3.3 Views

**FIGURE 2:**
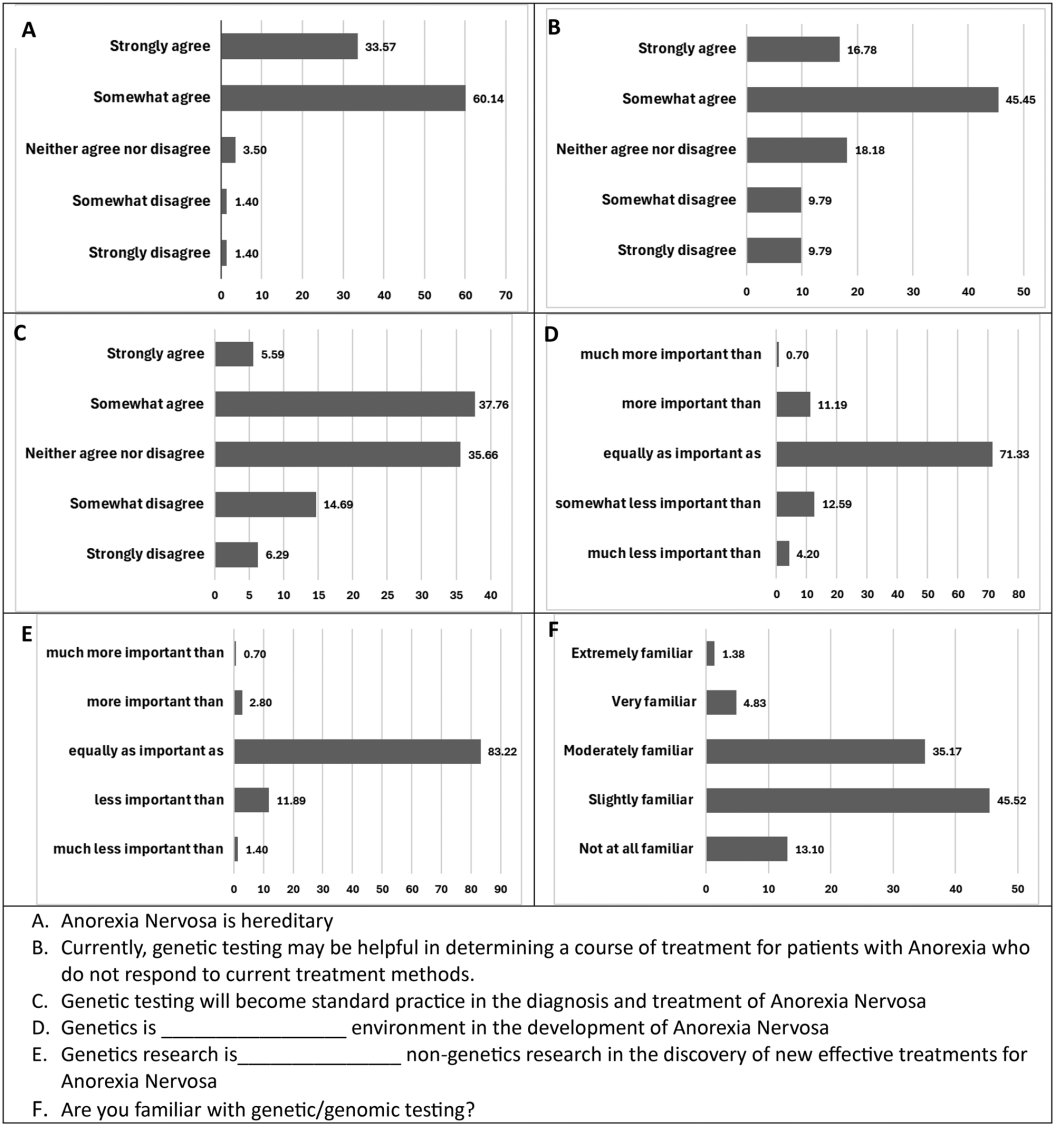
Respondent viewpoints and self‐assessed familiarity with genetic testing. Reponses to Questions A–F are shown. Numbers reflect the percentage of total participants responding as indicated.

To assess viewpoints on the influence of genetics on AN, and the value of genetic research and testing both currently and in the future, five Likert‐like statements were asked. Figure 2 provides a graphical representation of how respondents answered. Ninety‐three percent of respondents indicated that they either somewhat or strongly agreed with the statement “anorexia is hereditary,” 3.5% indicated that they neither agreed nor disagreed, and 2.8% indicated that they either somewhat or strongly disagreed. When asked about the influence of genetics and environment on the development of AN, 71.3% indicated that genetics and environment were equally important, 16.8% that genetics was somewhat or much less important, and 11.9% that genetics was more or much more important than environment in the development of AN. When asked about the importance of genetics research, 83.2% indicated that genetics research was as equally important as nongenetic research in the discovery of new treatments for AN, 13.3% indicated that genetics research was somewhat less or much less important, and 3.5% indicated that they believed genetics research was more or much more important than environmental influences research in finding new treatments for AN. Interestingly, though only 2.1% indicated that they currently offer genetic testing or genetic counseling to their patients, 62.2% of respondents indicated that they somewhat or strongly agreed with the statement “Currently, genetic testing may be helpful in determining a course of treatment for patients with anorexia who do not respond to current treatment methods,” and 43.4% indicated that they either somewhat or strongly believed that genetic testing will become standard practice in the diagnosis and treatment of AN in the future.

### 3.4 Relationships Between Data Groups

**FIGURE 3:**
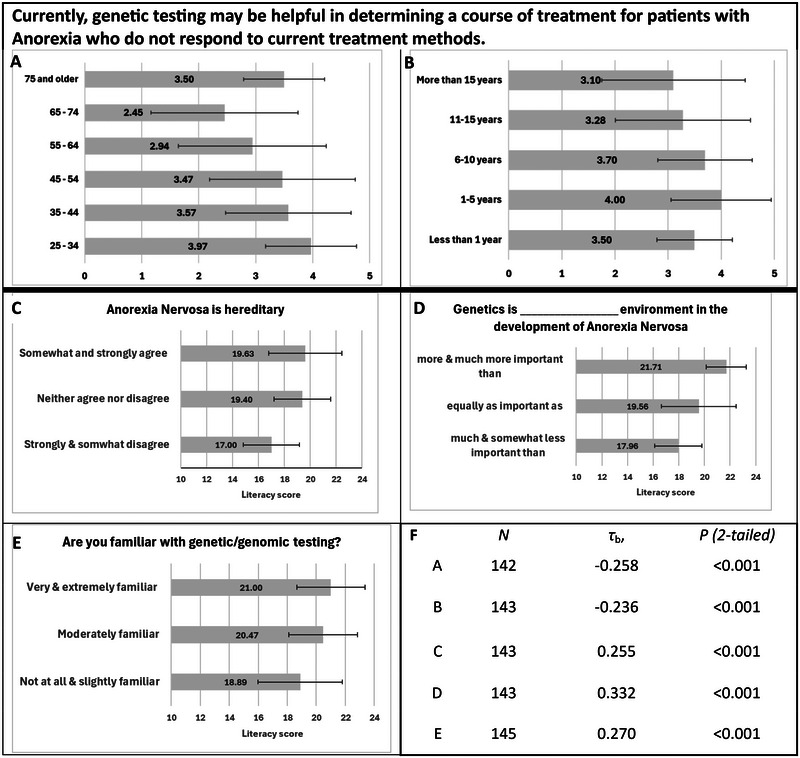
Significant correlations between literacy scores, demographics, and views responses. (A, B) Response to statement “Currently, genetic testing may be helpful in determining a course of treatment for patients with Anorexia who do not respond to current treatment methods” based on age (A) and years treating anorexia nervosa (B). *Strongly disagree* (1); *somewhat disagree* (2); *neither agree nor disagree* (3); *somewhat agree* (4); *strongly agree* (5). (C–E) Graphs depict the mean literacy scores and standard deviations for respondents in each group. (F) Statistics for (A)–(E). *τ*_b_ = calculated Kendall's tau‐b correlation coefficient.

Assessment of relationships between categorical, nominal demographic data, categorical ordinal Likert‐like data, and quantitative discrete numerical data was performed as described in Section 2. Genomic literacy was positively correlated with participants' self‐assessed genomics knowledge, and views on the influence of genetics on AN. Significant relationships are depicted graphically in Figure 3. Nonsignificant relationship data is found in Table S3. Not all participants answered every question, thus the *N* is provided for each calculation.

Participants' self‐assessed knowledge was positively correlated with their genomic literacy score. Those rating themselves as very or extremely familiar had higher GKnowM scores than those rating themselves as being only slightly or not at all familiar. Calculated *τ*_b_ = 0.270, *p* ≤ 0.001, *N* = 145.

GKnowM scores were also positively correlated with responses to heritability and genetic influence questions. Participants responding that they either strongly or somewhat disagreed with the statement “Anorexia nervosa is hereditary” had lower literacy scores than those responding that they either somewhat or strongly agreed. Calculated *τ*_b_ correlation coefficient, *τ*_b_ = 0.255, *p* ≤ 0.001, *N* = 143. With regard to genetic influence on the development of AN, participants responding that genetics is much or somewhat less important than environment in the development of AN had lower GKnowM scores than those stating that genetics is somewhat or much more important than environment. Calculated *τ*_b_ correlation coefficient, *τ*_b_ = 0.332, *p* ≤ 0.001, *N* = 143.

Statistically significant correlations were not identified between genomic literacy scores and answers to the following questions: “*Currently, genetic testing may be helpful in determining a course of treatment for patients with Anorexia who do not respond to current treatment methods*”; “*Genetic testing will become standard practice in the diagnosis and treatment of Anorexia Nervosa”*; and “*Genetics research is_______________ non‐genetics research in the discovery of new effective treatments for Anorexia Nervosa*”.

There was also no statistically significant difference identified between the age or years participants had been treating AN and literacy scores. However, there was a trend toward older and more experienced HCPs having higher genomic literacy scores. In addition, though the “MD/DO” group had the highest average literacy score (21.7, SD = 2.1), followed by the “PhD+” group (20.7, SD = 3.2), the RD+ group (19.7, SD = 2.9), the LPC+ group (19.1, SD = 2.5), the “other” group (17.7, SD = 2.1), and the one RN participant at 18, there was no significant difference found (ANOVA) (*p* = 0.07) (Figure S1).

There was a significant negative correlation identified between answers to the question “Currently, genetic testing may be helpful in determining a course of treatment for patients with anorexia who do not respond to current treatment methods” and age (calculated *τ*_b_ = −0.258, *p* ≤ 0.001, *N* = 142) and years treating AN (calculated *τ*_b_ = −0.236, *p* ≤ 0.001, *N* = 143). There was no significant correlation found between age or years treating AN and any of the other views questions (Table S3).

### 3.5 Thematic Analysis

**FIGURE 4:**
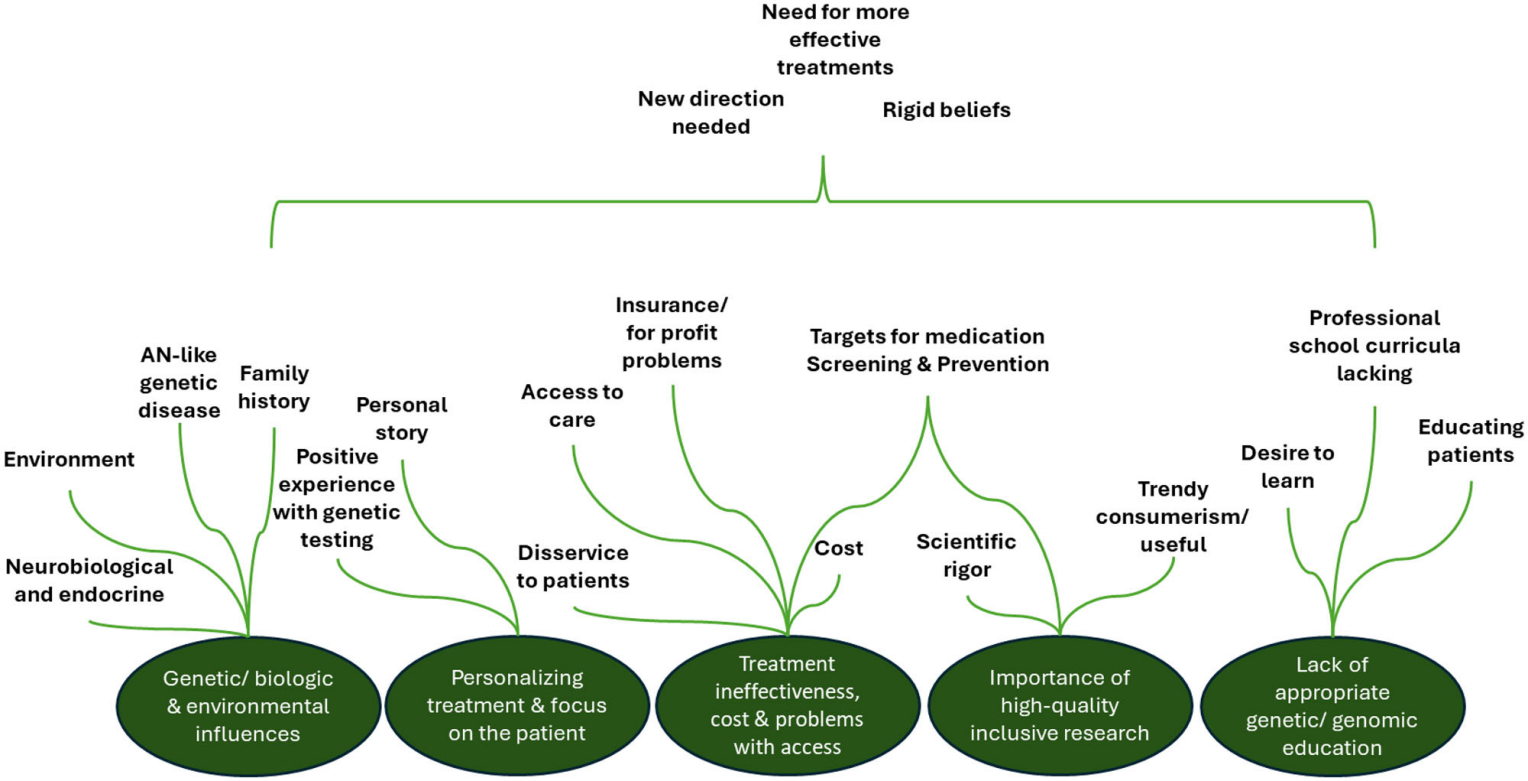
Thematic analysis map depicting the relationships between themes and subthemes identified in participant free‐text comments. See Table 2 for further detail.

Participants had the opportunity to provide feedback and additional information as free text at the end of the survey. Of the 145 participants, 26 (17.9%) also provided further thoughts and views. Thematic analysis was used to analyze this qualitative data. Table 2 provides an overview of the identified common codes and selected participant quotes. A thematic map is depicted in Figure 4.

**TABLE 2:** Thematic analysis of free text responses.

Codes	Number of participants contributing (*N* = 27)	Sample quote
Patients' family history of AN	4	“More often than not the people I treat have a family member (frequently aunt or mother) who also has struggled with AN.”
Need for genetics education/continuing education and desire to learn more	8	“My wish is for updated information on the genetics/genetic treatment of eating disorders to be in the curriculum and continuing education offerings for all clinicians who will inevitably encounter people with eating disorders.” “I would love to learn more about genetics and anorexia.”
Treatment for AN is lacking/needs improvement	11	“Considering how ineffective most treatments are for anorexia nervosa….” “…treatment efficacies for AN are of course generally poor”
Impact of for‐profit treatment centers	3	“My own care in 1984 was more thoughtful and effective that these for‐profit treatment programs now…” “In treatment centers, there is not usually any consideration of genetics and how we can use genetic information to help treat eating disorders, which I think is a disservice to the patients…”
Treatment of twins and patients with co‐occurring genetic illnesses	2	“I have clients with Type 1 diabetes and an eating disorder. I also have had several clients whom I have treated, as well as their twin or their sibling.”
Genetic testing and discussing genetics with patients	4	“It's amazing the difference I've seen patients have when they are able to see the genetic mutations that contribute to their disorder and find relief in more targeted treatments.” “What information would patients and families benefit most from learning about the genetics of AN?”
Current treatments and beliefs are rigid and lack personalization	3	“I feel it (genetic testing) will also better inform the medical community and hopefully, encourage health insurance companies to not see this as only a mental health condition.”
Importance of environment and other biological influences	4	“Though I believe eating disorders result from a combination of biological and environmental factors.” “I believe there may be an interaction but the development of AN is strongly related to the environment and trauma.” “I agree genetics research is very important, but I also hope for more neurobiological and endocrine‐related research with patients diagnosed with AN and other ED's.”
Balancing what is trendy/driven by consumerism with the needed scientific rigor needed when treating patients	3	“I sometimes worry about how services that become popular very quickly in the minds of the general public (or healthcare as a practice) start to be applied in ways that extend beyond available data and ultimately overshadow other effective assessment and treatment approaches.” “…there's a difference between what is useful, what is trendy, what insurance will pay for, and what people will be willing to do.” “While genetics has provided insights into the biological architecture of anorexia, it has not provided solid leads for treatment targets in therapy.”
Importance of screening and prevention	8	“If genetic testing can help with early intervention or response to intervention, it is a good thing!” “How can we use genetic screening in children to provide targeted prevention and early intervention programs for kids who are at high risk?” “I am very curious as to whether/how advancements in research on the genetic components of AN can improve our ability to prevent and/or treat the illness!!”
Importance of research/research participation	5	“Genetic research on anorexia does hold promise for identifying biological treatments and possible novel medication targets.” “I find that this research is pivotal in helping us understand the genetic risk of individuals with this condition.” “…I do think the way forward is likely going to be multidimensional and inclusive of multiple methodologies and approaches like genomic sequencing.”

There were five central themes identified: (1) lack of appropriate genetic/genomic education, (2) genetic/biologic and environmental influences, (3) personalizing treatment and focus on the patient, (4) importance of high‐quality, inclusive research, and finally, (5) treatment ineffectiveness, cost, and problems with access.

With regard to Theme 1, respondents expressed a desire to learn more about genetics and genomics. They also pointed to a lack of sufficient education and training in healthcare genetics both in graduate and medical school curricula and in continuing education. Patients were central in these remarks; responses included questions about how and what to communicate to those they treat.

Theme 2 was shaped by participants' expressed agreement that genetics plays a role in AN, but also remarks on the need to explore other biological influences, and the importance of environment in the development and persistence of AN. Respondents spoke of treating patients where there was a strong family history of AN, treating twins with AN, and treatment of patients with comorbid conditions, including genetic disorders and diabetes. This theme is tied into the third theme, which is the need for more personalized and targeted treatment. There was one mention of a case where genetic testing benefited a patient's progress. Some participants also provided personal stories of their experience with treatment before becoming healthcare practitioners.

Theme 4, the need to improve treatment effectiveness and provide more personalized and innovative care was the strongest theme, with the code “treatment for AN is lacking/needs improvement” recorded 11 times. Rigid beliefs around patient care and the need for a “new direction” were noted. Respondents also pointed out the influence that cost has on treatment, as well as insurance reimbursement. The final identified theme was the importance of high‐quality, inclusive research, and is closely tied to Theme 4. Respondents expressed the necessity of sound empirical evidence showing value before implementing “trendy” new testing or treatments.

## 4 Discussion

This study used a mixed methods approach to investigate the genomic literacy and viewpoints on the importance of genetics and genomics of those treating AN in the United States. Though the mean participant score was 19.6 (SD 2.8) (75% correct), this was slightly higher than the highest‐scoring HCP group studied by Linderman et al. (2021), suggesting that on average, the current study population had a better grasp of genetics and genomics than those included in the Linderman et al. survey. Overall, respondents were also able to assess their own knowledge level accurately.

The female majority of respondents reflects the fact that most of the HCPs treating eating disorders are female. In the United States, approximately 90% of dieticians (DataUSA 2024a), 76% of social workers (DataUSA 2024b), 72% of psychologists (DataUSA 2024c), and 39% of psychiatrists (Wyse et al. 2020) are female. As the first three of these groups comprised the majority of respondents, the level of female versus male respondents is not surprising.

Positive correlations were found between literacy and viewpoints on the heritability of AN as well as the importance of genetics research, suggesting that improving genomic literacy may increase HCP awareness of the strengths and limitations of genetics and genomics testing both now and in the future.

In the free text responses, HCPs expressed a desire to learn more about genetics and genomics but also pointed out a lack of learning opportunities both in training curricula and professional development. Respondents also communicated their frustration with current treatment options and effectiveness, especially for those with severe and enduring AN. On the other hand, HCPs noted the need for rigorous scientific evidence indicating that genetic testing could positively influence response to treatment before widespread use. Though not a focus of the current study, these responses reflect the realities of AN treatment in the United States today. Access to care is heavily influenced by the ability to pay. The data collected in this study also reflects the overall trend of HCPs leaving insurance networks due to reimbursement challenges and decreasing reimbursement rates, and the paucity of care available to rural patients (Benson et al. 2020; Benjenk and Chen 2020). Though a positive trend in telemedicine coverage by insurance companies coupled with more HCPs providing this modality of care may offset some of these gaps, further expansion, especially for low‐income and minority communities is needed (McBain et al. 2023).

### 4.1 Strengths and Limitations

This study is the first that the authors are aware of specifically assessing the genomic literacy and views on the importance of genetics of HCPs treating AN in the United States. The response group was diverse in age, years of experience, and area of practice as well as population served as assessed by location of practice (urban, suburban, and rural) and types of payment accepted.

The current study also had several limitations. First, though 145 HCPs responded, this only represents a small subset of those treating AN and a larger sampling may provide different results. Second, participants were primarily therapists and dieticians. A larger sampling and improved response rates from medical doctors and nurses would provide a clearer picture of differences in genomic literacy and views between the various educational backgrounds and therapeutic focus of HCPs treating AN in the United States.

### 4.2 Conclusions and Future Initiatives

This study provides a first look at the demographics and genomic literacy of a diverse subset of HCPs treating AN in the United States as well as their views on genetic testing and on the role that genetics plays in the development and persistence of AN. From the responses provided, there appears to be a lack of adequate and targeted genetics education for this group of professionals. Lower genomic literacy may impact views on the importance of genetics in the development of AN as well as genetic testing and research.

Some may see genetic education initiatives as being premature given that testing for the variants that have been identified as being associated with AN would not currently suggest a change in treatment approach. However, for those with SE‐AN, and atypical presentations, genetic testing to rule out other treatable conditions with similar phenotypes may be of value. Furthermore, patients are already having genetic testing done through DTC testing services, and it is important that HCPs can communicate what such testing can, and equally important, what it cannot predict. Recent studies have found that genetics education can improve HCPs’ comfort with genetics and genomics and confidence in the use of genetic testing (Scheuner et al. 2023; Hajek et al. 2022; Calabrò et al. 2021). Similar results would be expected for well‐developed and targeted genetic education for those treating AN.

As the clinical utility of AN PRS and other genetic assessments improve, it is reasonable to believe that they could be utilized with those at high risk for development of AN, for example individuals with a strong family history of AN and other eating disorders. In turn, preventative measures including increased attention to weight loss due to any cause and importance of subsequent regaining of weight could be implemented. For those already diagnosed with AN, testing could be used to identify those who are less likely to respond to traditional treatment. This could, in turn, provide further support for insurance coverage of biologically based and longer term treatment, thus reducing relapse rates and revolving door of treatment that currently exists.

## 5 Public Significance Statement

To the authors' knowledge, this study is the first to evaluate the genomic literacy and views on genetic testing of individuals treating AN in the United States. Findings support a relationship between the level of genomic knowledge and attitudes toward the contribution of genetics to the development of AN and the utility of genetic testing. The survey also identified a need for additional genetics and genomics educational opportunities.

## Author Contributions

**Sarah Ramsay**: conceptualization, research, writing–original draft. **Kendra Allison**: review and editing, second reviewer for thematic analysis. **William C. Bridges Jr**.: review of statistical approach and data. **Heide S. Temples**: review and editing. **Sara Sarasua**: review and editing.

## Conflicts of Interest

The authors declare no conflicts of interest.

### Peer Review

The peer review history for this article is available at https://publons.com/publon/10.1002/brb3.70441

## Supporting information




**Table 1**. Participants by state
**Table 2**: Percent correct answers for each of the GKnowM literacy test answers
**Table 3**. Associations between genomic literacy, demographics and views.
**Figure 1**. Mean Genomic literacy scores for different age groups, years treating anorexia nervosa and provider type.
**Figure 2**. Healthcare provider type of payment accepted for states that have and have not expanded Medicaid. States without expansion: Florida, Kansas, South Carolina, Tennessee, Texas, Wisconsin

## Data Availability

Non‐confidential data are available upon request from the corresponding author.

## Endnotes

1 The literature uses the terms genetic literacy and genomic literacy interchangeably. With the understanding that genetics refers to the study of genes and genomics is a more expansive term including the entire genome as well as interactions with environmental factors, the term genomic literacy is used herein.

2 The term healthcare provider is used herein to describe all those treating anorexia nervosa, including but not limited to medical doctors, therapists, nurses, and registered dieticians.
