# Supplementary material for: Genetic and Genomic Literacy of Healthcare Providers Treating Anorexia Nervosa in the United States: A Mixed Methods, Cross‐Sectional Study
Source: Brain Behav. 2025 Mar 31;15(4):e70441. doi: 10.1002/brb3.70441 (PMC11959152; doi:10.1002/brb3.70441)
Supplement: Supplementary file 1 — Table 1. Participants by state Table 2: Percent correct answers for each of the GKnowM literacy test answers Table 3. Associations between genomic literacy, demographics and views. Figure 1. Mean Genomic literacy scores for different age groups, years treating anorexia nervosa and provider type. Figure 2. Healthcare provider type of payment accepted for states that have and have not expanded Medicaid. States without expansion: Florida, Kansas, South Carolina, Tennessee, Texas, Wisconsin [file BRB3-15-e70441-s001.docx]

**Supplemental data 1:** Ramsay et al. Genetic and Genomic Literacy of Healthcare providers Treating Anorexia Nervosa in the United States: A mixed methods, cross sectional study

**Table 1.** Participants by state

| **In what state do you primarily practice?** | **Count of “In what state do you primarily practice?”** |
| --- | --- |
| Texas | 22 |
| North Carolina | 13 |
| New Jersey | 8 |
| California | 7 |
| Missouri | 7 |
| Virginia | 6 |
| Massachusetts | 6 |
| Washington | 6 |
| Arizona | 5 |
| Maryland | 5 |
| Florida | 4 |
| Tennessee | 4 |
| South Carolina | 4 |
| Pennsylvania | 4 |
| Wisconsin | 4 |
| Kansas | 3 |
| Colorado | 3 |
| New York | 3 |
| Illinois | 3 |
| Utah | 3 |
| New Hampshire | 3 |
| Ohio | 3 |
| Michigan | 2 |
| Minnesota | 2 |
| Iowa | 2 |
| Arkansas | 2 |
| Louisiana | 2 |
| Nevada | 2 |
| Vermont | 1 |
| Connecticut | 1 |
| Kentucky | 1 |
| Oregon | 1 |
| Oklahoma | 1 |
| Montana | 1 |
| Nebraska | 1 |
| **Grand Total** | **145** |

**Table 2**: Percent correct answers for each of the GKnowM literacy test answers

| **Question** | **Percent correct** |
| --- | --- |
| 1 | 59 |
| 2 | 85 |
| 3 | 99 |
| 4 | 92 |
| 5 | 91 |
| 6 | 90 |
| 7 | 96 |
| 8 | 100 |
| 9 | 65 |
| 10 | 86 |
| 11 | 99 |
| 12 | 69 |
| 13 | 62 |
| 14 | 84 |
| 15 | 88 |
| 16 | 56 |
| 17 | 76 |
| 18 | 76 |
| 19 | 58 |
| 20 | 34 |
| 21 | 79 |
| 22 | 74 |
| 23 | 86 |
| 24 | 44 |
| 25 | 44 |
| 26 | 67 |

**Table 3**. Associations between genomic literacy, demographics and views.

| Possible answers and numerical rating | |
| --- | --- |
| \| Strongly disagree (1) \| \| --- \| \| Somewhat disagree (2) \| \| Neither agree nor disagree (3) \| \| Somewhat agree (4) \| \| Strongly agree (5) \| | \| much less important than (1) \| \| --- \| \| somewhat less important than (2) \| \| equally as important as (3) \| \| more important than (4) \| \| much more important than (5) \| |

| **Literacy** | | | |
| --- | --- | --- | --- |
|  | ***τ*_b_** | ***p* (2-tailed)** | **N** |
| How many years have you been treating those with Anorexia Nervosa? | 0.085 | 0.194 | 145 |
| What is your age? | 0.058 | 0.374 | 144 |
| Genetic testing will become standard practice in the diagnosis and treatment of Anorexia Nervosa | 0.115 | 0.085 | 143 |
| Genetics research is_______________ non-genetics research in the discovery of new effective treatments for Anorexia Nervosa | 0.029 | 0.682 | 143 |
| Currently, genetic testing may be helpful in determining a course of treatment for patients with Anorexia who do not respond to current treatment methods. | -0.098 | 0.136 | 143 |

| **Age** | | | |
| --- | --- | --- | --- |
|  | ***τ*_b_** | ***p* (2-tailed)** | **N** |
| Anorexia Nervosa is hereditary | 0.105 | 0.155 | 142 |
| Genetics research is_______________ non-genetics research in the discovery of new effective treatments for Anorexia Nervosa | -0.063 | 0.396 | 142 |
| Genetics is _________________ environment in the development of Anorexia Nervosa | 0.049 | 0.497 | 142 |
| Genetic testing will become standard practice in the diagnosis and treatment of  Anorexia Nervosa | 0.048 | 0.496 | 142 |

| **How long treating Anorexia Nervosa** | | | |
| --- | --- | --- | --- |
|  | ***τ*_b_** | ***p* (2-tailed)** | **N** |
| Anorexia Nervosa is hereditary | 0.000 | 0.998 | 143 |
| Genetics research is_______________ non-genetics research in the discovery of new effective treatments for Anorexia Nervosa | -0.109 | 0.150 | 143 |
| Genetics is _________________ environment in the development of Anorexia Nervosa | 0.023 | 0.757 | 143 |
| Genetic testing will become standard practice in the diagnosis and treatment of  Anorexia Nervosa | 0.015 | 0.835 | 143 |

| **Mean Genomic Literacy Scores for Different Practitioner Types, Age Groups, and Years Treating Anorexia Nervosa** | **ANOVA** | | **Kendall’s** | |
| --- | --- | --- | --- | --- |
|  | ***P*** | **N** | ***τ*_b_** | ***p***  **(2-tailed)** |
| 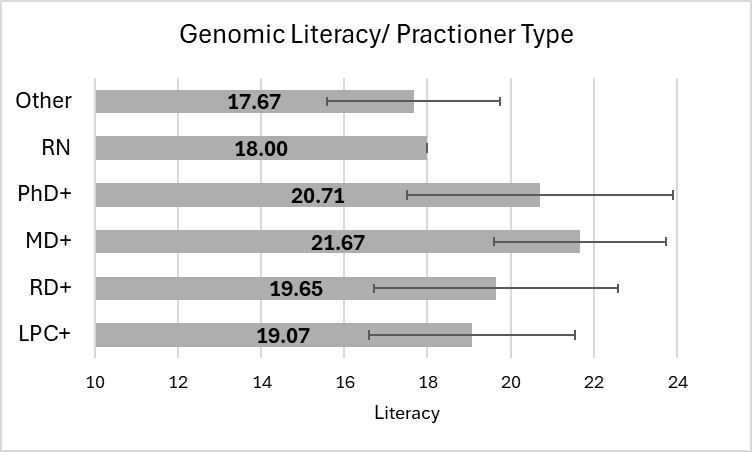 | 0.07 | 145 | N/A | N/A |
| 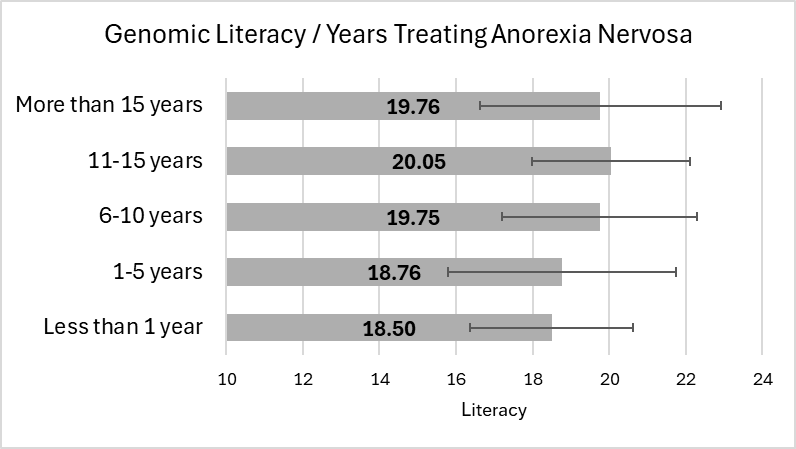 | N/A | 145 | 0.085 | 0.194 |
| 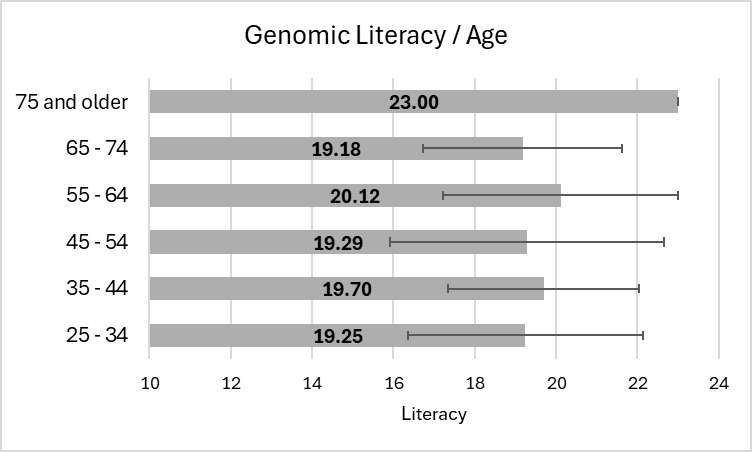 | N/A | 144 | 0.058 | 0.374 |

**Figure 1.**  Mean Genomic literacy scores for different age groups, years treating anorexia nervosa and provider type.

**Figure 2.** Healthcare provider type of payment accepted for states that have and have not expanded Medicaid. States without expansion: Florida, Kansas, South Carolina, Tennessee, Texas, Wisconsin
